# Insulin/IGF and sex hormone axes in human endometrium and associations with endometrial cancer risk factors

**DOI:** 10.1007/s10552-016-0751-4

**Published:** 2016-04-28

**Authors:** Melissa A. Merritt, Howard D. Strickler, Mark H. Einstein, Hannah P. Yang, Mark E. Sherman, Nicolas Wentzensen, Jurriaan Brouwer-Visser, Maria Jose Cossio, Kathleen D. Whitney, Herbert Yu, Marc J. Gunter, Gloria S. Huang

**Affiliations:** Department of Epidemiology and Biostatistics, School of Public Health, Imperial College London, St Mary’s Campus, Norfolk Place, London, W2 1PG UK; Department of Epidemiology and Population Health, Albert Einstein College of Medicine, Jack and Pearl Resnick Campus, 1300 Morris Park Avenue, Bronx, NY 10461 USA; Division of Gynecologic Oncology, Department of Obstetrics & Gynecology and Women’s Health, Albert Einstein College of Medicine and Montefiore Medical Center, 1300 Morris Park Avenue, Bronx, NY 10461 USA; Division of Cancer Epidemiology and Genetics, National Cancer Institute, 9609 Medical Center Drive, Bethesda, MD 20892 USA; Division of Cancer Prevention, National Cancer Institute, 9609 Medical Center Drive, Bethesda, MD 20892 USA; Department of Pathology, Jack D. Weiler Hospital, The University Hospital for Albert Einstein College of Medicine, Montefiore Medical Center, 1825 Eastchester Road, Room 338, Bronx, NY 10461 USA; Cancer Epidemiology Program, University of Hawaii Cancer Center, 701 Ilalo Street, Honolulu, HI 96813 USA

**Keywords:** Insulin-like growth factor, Insulin, Estrogen receptor, Endometrium, Endometrial cancer

## Abstract

**Purpose:**

Experimental and observational data link insulin, insulin-like growth factor (IGF), and estrogens to endometrial tumorigenesis. However, there are limited data regarding insulin/IGF and sex hormone axes protein and gene expression in normal endometrial tissues, and very few studies have examined the impact of endometrial cancer risk factors on endometrial tissue biology.

**Methods:**

We evaluated endometrial tissues from 77 premenopausal and 30 postmenopausal women who underwent hysterectomy for benign indications and had provided epidemiological data. Endometrial tissue mRNA and protein levels were measured using quantitative real-time PCR and immunohistochemistry, respectively.

**Results:**

In postmenopausal women, we observed higher levels of phosphorylated IGF-I/insulin receptor (pIGF1R/pIR) in diabetic versus non-diabetic women (*p* value =0.02), while women who reported regular nonsteroidal anti-inflammatory drug use versus no use had higher levels of insulin and progesterone receptors (both *p* values ≤0.03). We also noted differences in pIGF1R/pIR staining with OC use (postmenopausal women only), and the proportion of estrogen receptor-positive tissues varied by the number of live births and PTEN status (premenopausal only) (*p* values ≤0.04). Compared to premenopausal proliferative phase women, postmenopausal women exhibited lower mRNA levels of *IGF1*, but higher *IGFBP1* and *IGFBP3* expression (all *p* values ≤0.004), and higher protein levels of the receptors for estrogen, insulin, and IGF-I (all *p* values ≤0.02). Conversely, pIGF1R/pIR levels were higher in premenopausal proliferative phase versus postmenopausal endometrium (*p* value =0.01).

**Conclusions:**

These results highlight links between endometrial cancer risk factors and mechanistic factors that may contribute to early events in the multistage process of endometrial carcinogenesis.

**Electronic supplementary material:**

The online version of this article (doi:10.1007/s10552-016-0751-4) contains supplementary material, which is available to authorized users.

## Introduction

Endometrial cancer is the most common gynecologic malignancy worldwide with 319,605 new cases diagnosed in 2012 [1]. Obesity has been consistently associated with an increased risk of developing endometrial cancer [2, 3], and approximately 40 % of new cases in developed countries are thought to be attributable to a high body mass index (BMI ≥ 25 kg/m^2^) [4]. Other factors that are associated with a higher risk of endometrial cancer include a history of diabetes [5], postmenopausal estrogen only hormone use [6], an earlier age at menarche, later age of menopause, and nulliparity [7, 8]. Factors that appear to lower endometrial cancer risk include use of oral contraceptives (OCs) [7, 8] and aspirin but not other nonsteroidal anti-inflammatory drugs (NSAIDs) [9].

Few studies have examined the impact of endometrial cancer risk factors, such as obesity, diabetes, and nonuse of aspirin on the insulin/IGF and sex hormone axes in normal endometrial tissues. In an earlier study, Argenta et al. [10] examined hormone receptor expression in 46 obese women who underwent endometrial tissue sampling before and after bariatric surgery, and they observed similar patterns in estrogen receptor (ER) immunohistochemical (IHC) staining following surgery. Based on the same normal endometrium study population as the current study, Yang et al. [11] assessed the relationship between PTEN loss and exposure to endometrial cancer risk factors, and they reported that NSAID use was associated with PTEN loss while there was no difference in PTEN IHC staining for other risk factors.

The biological mechanisms that underlie the association of endometrial cancer with obesity, diabetes, and other risk factors are not well understood. The insulin-like growth factor (IGF) and sex hormone axes play important roles in endometrial physiology [12, 13], and studies mainly focusing on circulating insulin/IGF and sex hormone axes suggest that these pathways are considerably dysregulated in obesity and diabetes as well as in endometrial cancer development [14]. Higher estrogen levels (that are not simultaneously opposed by progesterone) have been associated with a higher risk of developing endometrial cancer [14, 15]. For example, in premenopausal women, ovarian hyperandrogenism may lead to progesterone deficiency, while in postmenopausal women, an increasing BMI has been linked to higher circulating estrogen levels with adipose tissue as the primary site of estrogen production from androgen precursors [16, 17]. In a prospective study of 124 postmenopausal endometrial cancer cases from three cohorts in New York, Northern Sweden, and Milan, there was a 4.1-fold increase in endometrial cancer risk for women in the top versus bottom quartile of estradiol levels [18]. Obesity is also associated with higher serum insulin levels [19], and several prospective cohort studies have reported positive associations between circulating insulin levels and endometrial cancer risk [20–22]. For example, among postmenopausal nonusers of hormone therapy, women who were classified in the highest versus lowest quartile of insulin levels had a 2.3-fold increased risk of developing endometrioid-type endometrial cancer [21].

In addition to the effects of circulating insulin/IGF levels, insulin/IGF receptor activation is also influenced by local tissue levels of IGF ligands as well as tissue levels of IGF-binding proteins via their regulation of ligand bioavailability. While insulin predominantly signals through the insulin receptor (IR), IGFs bind to the IGF-I receptor (IGF1R), as well as the IR, and hybrid IR/IGF1R receptors [19, 23]. There is extensive crosstalk between the sex hormone and insulin/IGF axes. The downstream mitogenic and anti-apoptotic effects of estrogen and insulin/IGF signaling converge on the AKT signaling pathway whose activation is suppressed by the phosphatase activity of the phosphatase and tensin homolog (PTEN) tumor suppressor. In endometrial cancer, loss of heterozygosity at the PTEN region has been reported in approximately 40 % of cases and somatic PTEN mutations have been identified in 37–83 % of tumors [24, 25]. Loss of PTEN function may represent an important early event in endometrial carcinogenesis, which may be mechanistically linked to loss of suppression of the mitogenic effects of estrogen, insulin, and growth factors [11, 26]. In the current study, we evaluated the tissue expression of IGF ligands (IGF1, IGF2), IGF-binding proteins 1 and 3 (IGFBP1 and IGFBP3), the tissue expression and activation of the insulin/IGF receptors [IR, IGF1R, phosphorylated (activated) IGF-I/insulin receptor (pIGF1R/pIR)], as well as the status of the hormone receptors [ER, progesterone receptor (PR)], and expression of PTEN in endometrial tissues from 77 premenopausal and 30 postmenopausal women who underwent hysterectomy for benign indications.

## Materials and methods

### Study population

The study population was comprised of women who underwent hysterectomy for benign indications such as uterine prolapse and fibroids. Participants were recruited from two studies, the Benign Reproductive Tissue Evaluation (BRTE) and Einstein Normal Endometrium (ENE) studies. The BRTE study included 150 consecutive mostly premenopausal women who underwent a hysterectomy at the Magee Women’s Hospital, Pittsburgh, Pennsylvania, USA, from 2006 to 2011 and who met the study’s eligibility criteria. Specifically, these subjects were required to be between the ages of 18–54 years, reported no use of exogenous hormones within three months of enrollment, and had not been diagnosed with any cancer when they had their hysterectomy [11]. The ENE study sequentially enrolled 50 postmenopausal women who consented to participate as they presented for hysterectomy to treat uterine prolapse at the Albert Einstein Hospital and Montefiore Medical Center, Bronx, New York, USA, between 2010 and 2014. Subjects could be of any age, but could not have any cancer at the time of their hysterectomy. Patients with uterine fibroids were eligible for the study, but only tissues that were not adjacent to the fibroids (when present) were sampled in case fibroid growth also interacts with these pathways. The BRTE and ENE studies only included subjects who did not have cancer at the time of hysterectomy; therefore, endometrial cancer tissue samples were unavailable for the current study. The current investigation included all participants who completed the required questionnaire, had known menopausal status and sufficient endometrial tissues for the studies; this resulted in a study population of 107 participants (*n* = 78 BRTE; *n* = 29 ENE). Informed consent was provided by all study participants, and the study was approved by the Institutional Review Board of the US National Cancer Institute and the Albert Einstein College of Medicine and Montefiore Medical Center.

### Tissue collection

Endometrial tissues were collected during hysterectomy surgery and immediately flash-frozen and/or fixed in 10 % buffered formalin to embed in paraffin. All tissues were frozen or formalin-fixed within 30–60 min of operative removal when possible.

### Lifestyle questionnaire

All participants completed a self-administered study-specific questionnaire that included questions on general medical history, reproductive history (e.g., parity, ages at menarche, and menopause), use of OCs and postmenopausal hormone therapies, smoking, height, weight, and NSAID use. NSAID use was defined as any use of aspirin or ibuprofen in the past 12 months (BRTE) or regular use of aspirin, acetaminophen, or other anti-inflammatory drugs such as Ibuprofen, Naproxen, Piroxicam, Indomethacin, Sulindac, or COX-2 inhibitors, e.g., Celecoxib or Rofecoxib (ENE). In analyses of aspirin versus non-aspirin NSAIDs, very few participants were exclusive aspirin users. Therefore, the aspirin user category included participants who reported any aspirin use (with or without non-aspirin NSAID use). All of the ENE study participants were postmenopausal at enrollment. In the BRTE study, participants were asked about their menstrual cycle pattern, and they were classified as premenopausal if they reported regular or irregular monthly periods in the 12 months prior to enrollment.

### Laboratory assays

IHC staining was carried out for: IGF1R; IR; pIGF1R/pIR; ER α; and PR. IHC staining was performed on 5-µm sections of formalin-fixed paraffin-embedded endometrial tissues from 106 participants (*n* = 78 BRTE; *n* = 28 ENE). Tissue sections were deparaffinized using xylene and graded alcohols. Antigen retrieval was performed in Target Retrieval Solution, pH 6.1 (Dako, Inc., Carpinteria, CA, USA) at 95 °C for 30 min. After cooling at room temperature, slides were rinsed in Tris-buffered saline (TBS), pH 7.5 (0.02 M Tris/Tris-HCl and 0.15 M NaCl). Endogenous peroxidase was quenched with peroxidase blocking reagent (Dako, Inc.) for 10 min. After rinsing with TBS, blocking solution was applied for 30 min at room temperature, and then the primary antibody solution was applied. IHC conditions for the primary antibodies are summarized in Supplementary Table 1. Slides were washed three times with TBS-T (0.1 % Tween 20), then incubated with the appropriate secondary antibody (EnVision™ + Kits, Dako, Inc.). After 3× washes with TBS-T, slides were incubated with diaminobenzidine chromogen solution. Slides were rinsed with water and counterstained with hematoxylin, dehydrated through graded alcohols, absolute ethanol, and xylene, then coverslipped with mounting medium. Positive control tissues and negative control slides were stained in parallel for all IHC assays.

The slides were scored by the study pathologist (K.W.). IHC staining scores were estimated separately for glandular and stromal cells. For IGF1R, pIGF1R/pIR, and IR, both nuclear and cytoplasmic cellular localizations were evaluated, while for ER and PR only nuclear staining was assessed. Nuclear localization of the IR and IGF1R has been observed previously and may have functional significance [27]. PTEN immunostaining was measured for 81 participants (*n* = 65 BRTE; *n* = 16 ENE) using a validated monoclonal antibody that can detect PTEN loss (i.e., PTEN-null, or loss of PTEN protein expression in microscopically normal endometrial glands) [28] (Supplementary Table 1). PTEN staining was scored by a second study pathologist (M.E.S.) who at the same time reviewed the slides to classify the menstrual cycle phase (proliferative, secretory, or other) for the BRTE cases at their time of surgery.

Gene expression (mRNA levels) of *IGF1*, *IGF2*, *IGFBP1,* and *IGFBP3* was measured with qPCR using investigator-validated primers (Supplementary Table 2) in the subset of patients with adequate frozen tissues available (i.e., good quality RNA was obtained) (*n* = 37 BRTE; *n* = 26 ENE). To extract RNA, whole frozen tissues were pulverized in a tissueTUBE bag (Covaris, Woburn, MA, USA) using a Covaris CryoPrep and then homogenized in Buffer RLT (Qiagen, Valencia, CA, USA) using a Covaris adaptive focused acoustics tissue disrupter. The Qiagen AllPrep kit was used following the manufacturer’s instructions for isolation of RNA and DNA. The RNA concentration and purity was measured using a Nanodrop spectrophotometer (NanoDrop Technologies, Wilmington, DE, USA), and RNA integrity was evaluated with the Agilent Bioanalyzer (Agilent, Santa Clara, CA, USA). RNA quality was uniformly excellent and met the following criteria: Nanodrop, 260/280 ratio >1.8; Agilent Bioanalyzer, RIN > 6. Following RNA extraction and purification, complementary DNA was synthesized from 1 μg of total RNA using the SuperScript VILO cDNA Synthesis Kit (Life Technologies, Grand Island, NY, USA). Quantitative real-time PCRs were carried out using investigator-validated primers for the target genes [29] (Supplementary Table 2), and PowerSYBR Green (Life Technologies) detection according to the manufacturer’s recommendations. The reaction scale was adjusted for use in 384-well plates on the Applied Biosystems 7900HT Fast Real-Time PCR System (Life Technologies). Target gene expression was internally normalized to the expression of the housekeeping gene *peptidylprolyl isomerase B* (*PPIB*), and each reaction was run in triplicate on the same plate. Each assay plate included two reactions that omit either the mRNA template or the reverse transcriptase enzyme to exclude the possibility of contamination. RNA concentrations were provided as raw Ct values, and expression scores were calculated using 2^(−deltaCt) × 1,000 [arbitrary units/scaling factor] [30].

### Statistical analysis

For IHC data, the staining intensity in different areas of the section was assessed using standardized ranges and allocated a value of 0 (none), 1 (weak), 2 (moderate), or 3 (strong). The percentage of cells that stained positive in five 40× fields was estimated, and the percentage of positive cells was multiplied by the intensity value to calculate the H-score with a maximum value of 300. For ER and PR staining, a cutoff H-score ≥75 was used to differentiate between positive and negative staining as previously described [31]. For consistency with a previous study [32], endometrial tissues were classified as positive for IGF1R, pIGF1R/pIR, and IR staining if the H-score was >20 (equivalent to a 2+ staining intensity in >10 % positive cells). PTEN staining was scored as wild type (PTEN present) or PTEN-null.

We evaluated measures of protein and gene expression levels in endometrial tissues across binary categories of endometrial cancer risk factors as follows: BMI (dichotomized at the median, 28.3 kg/m^2^), self-reported diabetes (no, yes), smoking status (never smoked, former/current smoker), ever pregnant (no, yes), number of children among women with ≥1 live birth (1–2, ≥3), OC use (never, ever), age at menarche (age <12 years, ≥12), any NSAID use (no, yes), and NSAID type (among ever NSAID users; aspirin, non-aspirin only). To avoid possible confounding by menopausal status and study site, all risk factor comparisons were restricted to premenopausal or postmenopausal subjects. For premenopausal BRTE participants, protein or gene expression levels were additionally examined in relation to menstrual cycle phase (proliferative, secretory) at the time of hysterectomy. In the comparison by menopausal status, we evaluated premenopausal (proliferative phase only) versus postmenopausal women in order to assess insulin/IGF and sex hormone axes in a more uniform premenopausal group who were likely exposed to higher estrogen levels at the time of their hysterectomy.

We tested whether there were differences in protein levels in endometrial tissues according to risk factor categories using Fisher’s exact tests, while gene expression levels (assessed continuously) were compared across risk factor categories using Wilcoxon tests. Analyses were only undertaken if there were *n* ≥ 5 samples in each comparison category. All analyses were carried out separately according to menopausal status. Statistical tests were two-sided, and a *p* value <0.05 was considered statistically significant. Statistical analyses were performed using R version 3.1.1 [33].

## Results

The BRTE and ENE studies included mostly premenopausal and postmenopausal participants, respectively; therefore, BRTE participants were younger (BRTE mean age = 43.4 years vs. ENE mean = 60.9 years) (Table 1). BRTE versus ENE participants, respectively, were more likely to have ever used OCs (73 vs. 48 %) and NSAIDs (69 vs. 48 %), had a longer duration of OC use (8.9 years vs. 5.2), and were more likely to be current/former smokers (50 vs. 21 %). The proportion of ever-pregnant women was similar across studies; however, BRTE participants had fewer live births (BRTE mean = 1.8 live births vs. ENE mean = 2.6).

**Table 1:** Characteristics of the BRTE and ENE study populations

Study	BRTE (*n* = 78)	ENE (*n* = 29)
Age (years)^a^, mean (SD)	43.4 (6.0)	60.9 (8.0)
Duration OC use^a,b^ (years), mean (SD)	8.9 (6.0)	5.2 (3.6)
Number of live births^c^, mean (SD)	1.8 (1.0)	2.6 (1.3)
Age at menarche^a^ (years), mean (SD)	12.5 (1.8)	11.7 (1.6)
Body mass index^a^ (kg/m^2^), mean (SD)	29.4 (6.5)	29.3 (5.8)
Ever-use OCs^a,d^, *n* (%)	56 (73)	14 (48)
Ever pregnant^e^, *n* (%)	68 (87)	26 (90)
Premenopausal, *n* (%)	77 (99)	0
Postmenopausal, *n* (%)	1 (1)	29 (100)
Postmenopausal hormone use, *n* (%)	–	0
Any NSAID use^a^, *n* (%)	53 (69)	13 (48)
Current/former smoker, *n* (%)	39 (50)	6 (21)
Indication for hysterectomy		
Adenomyosis, *n* (%)	5 (6)	0
Leiomyomata/fibroids, *n* (%)	25 (32)	3 (10)
Uterine prolapse, *n* (%)	1 (1)	24 (83)
Endometriosis, *n* (%)	12 (15)	0
Abnormal uterine bleeding, *n* (%)	9 (12)	0
1+ above indications, *n* (%)	12 (15)	1 (3)
Other reason/missing, *n* (%)	14 (18)	1 (3)

^a^Missing data were ≤2.9 %

^b^Among ever users of OCs (use ≥1 year)

^c^Among parous women

^d^Ever-use OCs defined as use ≥1 year

^e^Ever pregnant includes live births, still births, and miscarriages

### Evaluation of endometrial cancer risk factors in relation to endometrial tissue protein and mRNA levels of insulin/IGF and sex hormone axes

**Fig. 1:**
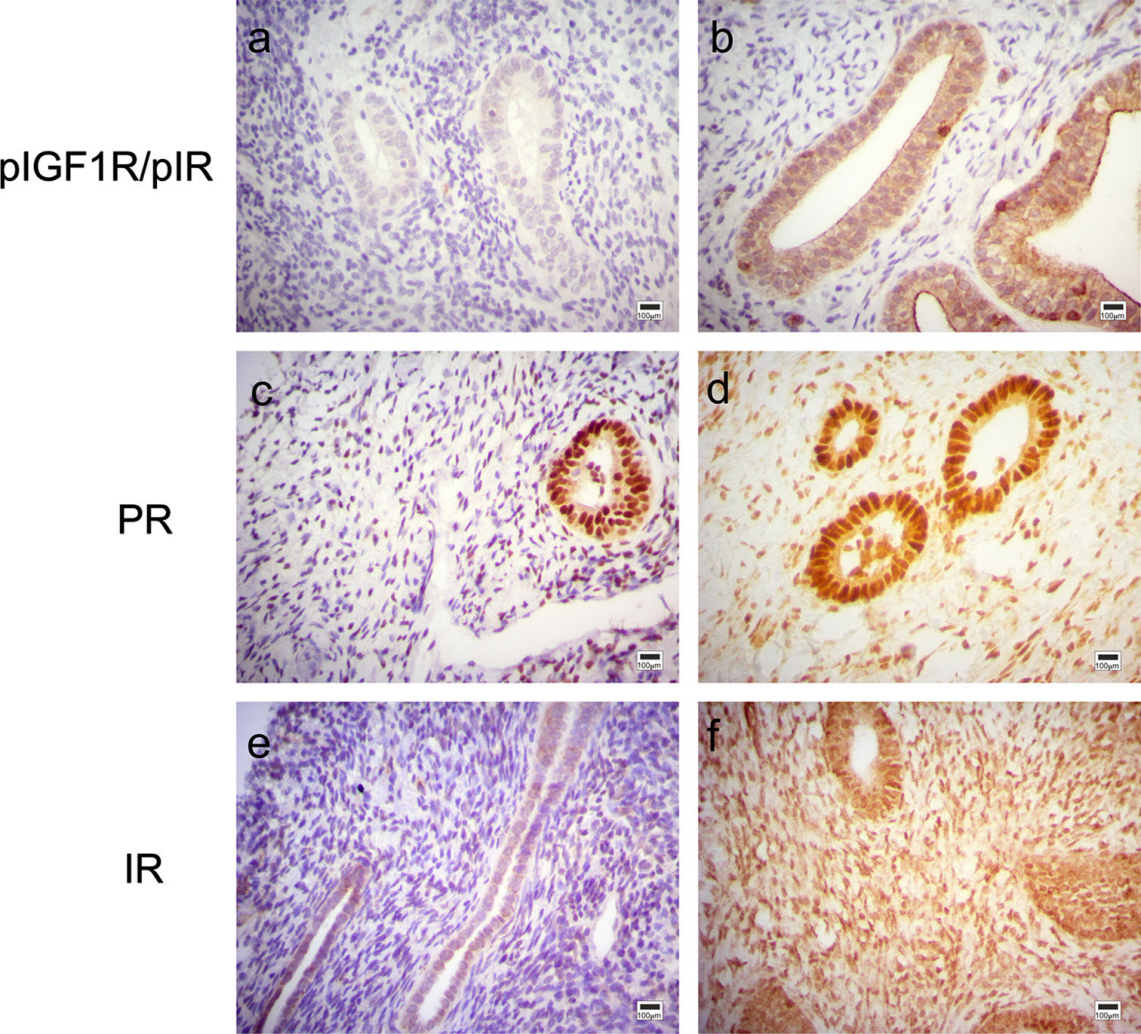
Insulin/IGF and sex hormone axes protein expression in endometrium in relation to diabetes status and NSAID use. **a** pIGF1R/pIR glandular cytoplasmic staining is negative in this representative endometrial tissue sample from a non-diabetic postmenopausal patient. **b** pIGF1R/pIR glandular cytoplasmic staining is positive in this representative endometrial tissue sample from a diabetic postmenopausal patient. **c** PR stromal nuclear staining is low in this representative endometrial tissue sample from a postmenopausal patient who reported no NSAID use. This tissue also shows positive nuclear PR glandular staining. **d** PR stromal nuclear staining is high in this representative endometrial tissue sample from a postmenopausal NSAID user. This tissue also shows positive nuclear PR glandular staining. **e** IR stromal nuclear staining was negative in this representative endometrial tissue sample from a postmenopausal patient who reported no NSAID use. **f** IR stromal and glandular nuclear staining was strongly positive in this representative endometrial tissue sample from a postmenopausal NSAID user. All images shown are of equal magnification (×400) and scale (100-µm *scale bar* is pictured)

We examined endometrial cancer risk factors, namely diabetes, BMI, smoking, NSAID use and type, age at menarche, parity, OC use, and PTEN status in relation to protein and gene expression levels in endometrial tissues. There were significant differences in protein levels for several of the risk factor comparisons; for example, postmenopausal participants with diabetes had a higher frequency of positive pIGF1R/pIR endometrial IHC staining as compared to non-diabetics, respectively [pIGF1R/pIR glandular cytoplasmic staining, 6/7 (86 %) positive vs. 5/18 (28 %) positive, *p* value = 0.02] (Table 2; Fig. 1a, b). In the endometrium of postmenopausal NSAID users, we observed that a higher proportion of subjects had positive IHC staining for PR and IR, respectively, than nonusers of NSAIDS [PR stromal staining, 13/13 (100 %) positive vs. 7/13 (54 %), *p* value =0.01, Fig. 1c, d; IR stromal nuclear staining, 12/13 (92 %) positive vs. 6/13 (46 %), *p* value =0.03, Fig. 1e, f]. Among parous premenopausal participants, a higher proportion of women with 3+ live births had ER-positive glandular cell staining (9/11 (82 %) positive) versus women with 1–2 children (15/34 (44 %) positive, *p* value =0.04). In postmenopausal endometrial tissues, there was a higher frequency of pIGF1R/pIR nuclear glandular cell staining in OC users than in nonusers, respectively (8/13 (62 %) positive vs. 2/13 (15 %), *p* value =0.04). Lastly, we observed that a higher proportion of participants had ER-positive glandular cell staining in premenopausal endometrial tissues that were classified as PTEN-null [8/8 (100 %) ER positive] versus PTEN wild-type [21/44 (48 %) ER positive, *p* value =0.01]. For the above-mentioned results, the IHC staining patterns were restricted to either premenopausal or postmenopausal tissues. Due to an insufficient number of cases, we were unable to analyze diabetes in premenopausal women and PTEN status and ever versus never pregnant in postmenopausal women. For other proteins, the frequency of positive/negative IHC staining did not vary according to diabetes, NSAID use, parity, OC use, and PTEN status, and none of the IHC proteins for any insulin/IGF or sex hormone axis components differed in comparisons of BMI, smoking status, age at menarche, ever versus never pregnant, and NSAID type (Supplementary Tables 3 and 4).

**Table 2:** Immunohistochemical staining of insulin/IGF and sex hormone axes in endometrium in relation to endometrial cancer risk factors

	Premenopausal (*n* = 77)	Postmenopausal (*n* = 29)
*pIGF1R/pIR-c (gland)*		
Diabetes no	26/59 (44)	5/18 (28)
Diabetes yes	N/A	6/7 (86)
*p* value^a^	–	**0.02**
*pIGF1R/pIR-n (gland)*		
OC use no^b^	3/16 (19)	2/13 (15)
OC use yes^b^	9/42 (21)	8/13 (62)
*p* value^a^	1.00	**0.04**
*ER (gland)*		
Live births^c^ 1–2	15/34 (44)	13/13 (100)
Live births^c^ 3+	9/11 (82)	10/12 (83)
*p* value^a^	**0.04**	0.22
*ER (gland)*		
PTEN wild-type	21/44 (48)	14/14 (100)
PTEN-null	8/8 (100)	2/3 (67)
*p* value^a^	**0.01**	–
*IR-n (stroma)*		
NSAID use no	17/22 (77)	6/13 (46)
NSAID use yes	33/44 (75)	12/13 (92)
*p* value^a^	1.00	**0.03**
*PR (stroma)*		
NSAID use no	7/8 (88)	7/13 (54)
NSAID use yes	12/15 (80)	13/13 (100)
*p* value^a^	1.00	**0.01**

Bold values indicate *p* < 0.05

*c* cytoplasmic, *ER* estrogen receptor alpha, *IR* insulin receptor, *n* nuclear, *N/A* none available, *pIGF1R/pIR* phosphorylated IGF1R/insulin receptor, *PR* progesterone receptor

Numbers in table refer to *n* positive/*n* total (% positive)

Numbers may not sum to total due to missing data on staining and/or the variable

^a^ *p* value from Fisher’s exact test

^b^Ever use of OCs defined as use ≥1 year

^c^Restricted to women who had 1+ live births

We did not observe differences in IGF axis gene expression levels in comparisons of the risk factor categories (data not shown); however, selected comparisons could not be carried out due to limited numbers (diabetes in premenopause, ever versus never pregnant in postmenopause, or PTEN status irrespective of menopausal status). We also were unable to evaluate protein and gene expression levels across the risk factor categories when stratifying by menstrual cycle phase among premenopausal women due to the limited sample size.

### Comparison of endometrial tissue protein and mRNA levels for insulin/IGF and sex hormone axes by menopausal status

**Fig. 2:**
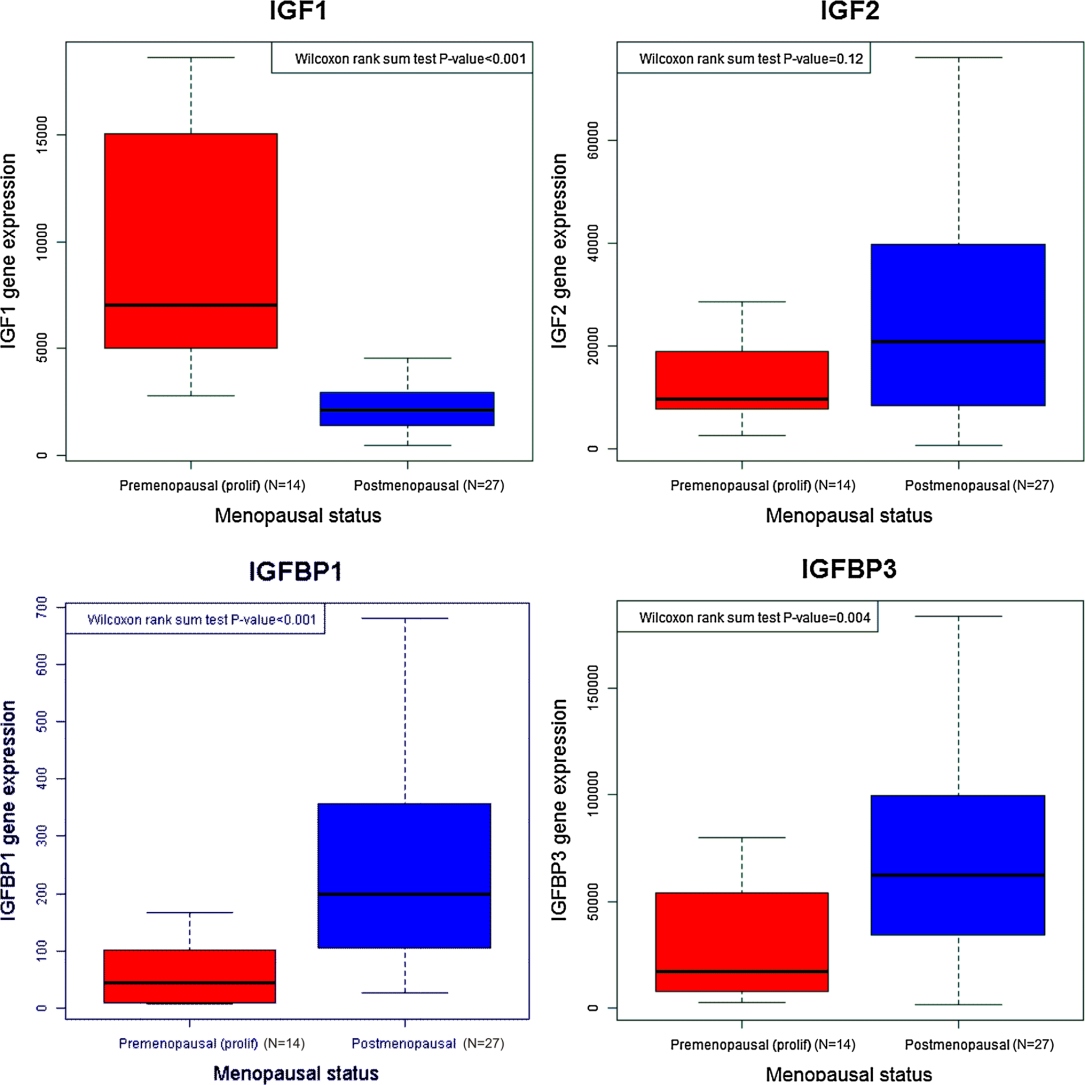
Gene expression of IGF axis genes in endometrium in relation to menopausal status. Gene expression values (normalized to *PPIB*) as detected by qPCR are pictured. *Box* and *whisker plots* depict the median (*line*), interquartile range (*box*), and *error bars* demonstrate the full range of the data

In endometrial tissues, a higher proportion of subjects had ER-positive staining by IHC in postmenopausal relative to proliferative phase premenopausal participants, respectively, for glandular tissue [26/29 (90 %) positive vs. 13/23 (57 %)] and in the stroma [21/29 (72 %) positive vs. 9/23 (39 %)], with both *P* values ≤0.02, whereas for PR (glandular or stromal tissue) there was no difference in the frequency of positive staining by menopausal status (Table 3). A higher proportion of postmenopausal women versus proliferative phase premenopausal participants, respectively, had positive glandular cytoplasmic staining for the IR [25/28 (89 %) positive vs. 13/27 (48 %), *p* value =0.001] and glandular nuclear staining for the IGF1R [18/28 (64 %) positive vs. 4/26 (15 %), *p* value <0.001]. In contrast, there was no difference in glandular nuclear staining for the pIGF1R/pIR, and a lower proportion of postmenopausal versus proliferative phase premenopausal participants, respectively, had positive pIGF1R/pIR stromal nuclear staining [4/26 (15 %) positive vs. 12/23 (52 %), respectively, *p* value =0.01]. There were also significant differences in IGF axis mRNA levels in endometrial tissues by menopausal status; compared with proliferative phase premenopausal women, postmenopausal women had lower expression of *IGF1* and higher expression of *IGFBP1* and *IGFBP3*, whereas *IGF2* gene expression levels did not differ by menopausal status (Fig. 2; Supplementary Table 5).

**Table 3:** Immunohistochemical staining of insulin/IGF and sex hormone axes in premenopausal and postmenopausal endometrium

Protein	Premenopausal (all subjects) (*n* = 77)	Premenopausal (proliferative) (*n* = 29)	Postmenopausal (*n* = 29)	*p* value^a^	*p* value^b^
ER (gland)	32/58 (55)	13/23 (57)	26/29 (90)	0.001	0.009
ER (stroma)	19/58 (33)	9/23 (39)	21/29 (72)	0.001	0.02
PR (gland)	20/24 (83)	8/9 (89)	26/28 (93)	0.40	1.00
PR (stroma)	19/23 (83)	8/9 (89)	22/28 (79)	1.00	0.66
IR-c (gland)	38/66 (58)	13/27 (48)	25/28 (89)	0.003	0.001
IR-c (stroma)	17/66 (26)	6/27 (22)	2/28 (7)	0.05	0.14
IGF1R-n (gland)	9/62 (15)	4/26 (15)	18/28 (64)	<0.0001	<0.001
IGF1R-n (stroma)	11/62 (18)	5/26 (19)	10/28 (36)	0.10	0.23
pIGF1R/pIR-n (gland)	13/59 (22)	8/24 (33)	10/26 (38)	0.18	0.77
pIGF1R/pIR-n (stroma)	25/58 (43)	12/23 (52)	4/26 (15)	0.01	0.01

*ER* estrogen receptor alpha, *IGF1R* insulin-like growth factor 1 receptor, *c* cytoplasmic, *n* nuclear, *pIGF1R/pIR* phosphorylated IGF1R/phosphorylated insulin receptor, *IR* insulin receptor

Numbers in table refer to *n* positive/*n* total (% positive)

Numbers may not sum to total due to missing data on immunohistochemical staining

^a^ *P* value from Fisher’s exact test for the comparison of premenopausal (all subjects) versus postmenopausal

^b^ *P* value from Fisher’s exact test for the comparison of premenopausal (proliferative phase subjects) versus postmenopausal

In comparison with premenopausal women in the secretory menstrual cycle phase, there was a higher proportion of positive IR and pIGF1R/pIR protein expression in premenopausal proliferative phase tissues [IR glandular nuclear staining, 26/27 (96 %) positive vs. 18/24 (75 %), *p* value =0.04 (data not shown); pIGF1R/pIR glandular cytoplasmic staining, 15/24 (63 %) positive vs. 6/22 (27 %), *p* value =0.02] (Supplementary Figure 1 a, b). In contrast, we observed no difference in pIGF1R/pIR stromal cytoplasmic staining or glandular/stromal nuclear pIGF1R/pIR staining when comparing the proliferative versus secretory phase endometrium (data not shown). There also were no significant differences in the proportion of positive IHC staining for other assayed proteins in comparisons of menstrual cycle phase. In analyses of IGF axis mRNA levels, we observed suggestively higher levels of *IGFBP1* and *IGF2* in secretory versus proliferative phase premenopausal tissues (each *p* value =0.06, Supplementary Figure 2).

## Discussion

In this study of normal (benign) endometrial tissue in which expression of the insulin/IGF and sex hormone axes was evaluated in relation to endometrial cancer risk factors, we observed that among postmenopausal women there was a higher frequency of positive pIGF1R/pIR endometrial glandular cell cytoplasmic IHC staining in diabetic as compared with non-diabetic women. We also observed that a larger proportion of postmenopausal OC users versus OC nonusers had positive pIGF1R/pIR glandular cell nuclear staining. A higher proportion of regular NSAID users had positive staining for PR and IR than nonusers of NSAIDs. Among premenopausal participants, we noted that a higher frequency of ER-positive glandular cell staining in endometrial tissues from parous women with ≥3 live births versus 1–2 births and in PTEN-null versus wild-type endometrium.

Our observation of a higher frequency of pIGF1R/pIR endometrial tissue IHC staining in diabetic versus non-diabetic postmenopausal women could reflect the high levels of insulin in circulation among diabetic women, or increased bioactive IGF-I levels that might be induced by the reduction of IGFBP1 in circulation due to insulin-related downregulation of IGFBP1 production. We caution that this result was based on a small number of diabetic women and this finding therefore requires confirmation in larger studies that have the ability to examine insulin-resistant women who have not yet developed diabetes and to account for the possible effects of diabetes treatment on the endometrium since this information was unavailable in the current study. Nevertheless, this observation suggests a mechanistic link between diabetes, an established risk factor for endometrial cancer, and endometrial tumorigenesis. The pIGF1R/pIR pathway is of particular interest in relation to endometrial cancer development, and at least one previous study reported upregulation of pIGF1R/pIR in complex atypical endometrial hyperplasia, a putative precursor lesion for endometrial cancer, as well as in grade 1 endometrial cancers as compared with normal endometrium [32]. In contrast, we observed that OC users, who are expected to have a lower risk of developing endometrial cancer, also had a higher frequency of positive pIGF1R/pIR staining. It was notable that the pIGF1R/pIR glandular cell staining localizations differed for the diabetes (cytoplasmic staining) and OC use (nuclear staining) results. The cytoplasmic staining of pIGF1R/pIR in the endometrium of diabetic women is consistent with the well-characterized function of IGF1R which is that it becomes autophosphorylated upon ligand binding whereby its kinase activity leads to phosphorylation of its downstream substrates; subsequently, the activated receptor is internalized to the cytoplasm and recycled to the membrane [34]. In contrast, the role of nuclear pIGF1R/pIR is not well established, and its functional implication remains uncertain. However, the ability of the IGF1R and IR to translocate to the nuclear compartment has been previously reported [35]. The finding of a higher frequency of positive pIGF1R/pIR nuclear staining in OC users and its possible role in OC protection from endometrial cancer is a novel finding that warrants further investigation. These studies included a small number of postmenopausal endometrial samples; thus, our analyses are exploratory and require confirmation in additional studies.

In the current analysis, we observed that postmenopausal NSAID users had higher endometrial expression of PR but not ER. These findings are of interest as a recent meta-analysis of nine studies reported that any use of aspirin NSAIDs versus no use of any type of NSAIDs was associated with a 13 % lower risk of developing endometrial cancer [9]. Higher PR levels would presumably be protective for endometrial cancer development wherein progesterone inhibits the proliferation of endometrial epithelial cells via PR in stromal cells [36]. As far as we are aware, this study was the first to investigate NSAID use in relation to endometrial PR and ER levels; these findings warrant further study as NSAID and similar drug use is very common in this age group and might contribute to their potentially protective influence on endometrial cancer development. To our knowledge this was the first study to report differences in IHC staining patterns for pIGF1R/pIR with OC use and IR with NSAID use in postmenopausal women, as well as differences in ER staining by the number of live births and PTEN status in premenopausal women. This was an exploratory study with a limited number of tissue samples; therefore, the findings may be due to chance and these results require confirmation in additional studies.

It was notable that differences in IHC staining for selected risk factors were only observed in postmenopausal or premenopausal subjects but not across both subgroups. Although the number of postmenopausal versus premenopausal subjects was small, there were more significant findings in postmenopausal women; this may be because postmenopausal women are a more homogeneous group with respect to their hormone levels, and all of these women were nonusers of postmenopausal hormones. By comparison, tissues from premenopausal women were collected at different phases of the menstrual cycle and several components of the insulin/IGF and sex hormone axis vary by menstrual cycle phase as discussed below. Due to the exploratory nature and small sample size of this study, we cannot conclude that the findings are restricted to only postmenopausal or premenopausal women and this will require confirmation in further studies.

We also examined differences in key insulin/IGF and sex hormone axis components in endometrial tissues by menopausal status. In postmenopausal relative to proliferative phase premenopausal endometrium, there was a higher proportion of positive ER, glandular IR, and IGF1R IHC staining, and higher levels of *IGFBP1* and *IGFBP3* gene expression. In contrast, in proliferative phase premenopausal endometrium there was a higher frequency of positive pIGF1R/pIR staining (stromal cells only) and higher *IGF1* gene expression levels as compared with postmenopausal tissues. Among premenopausal women, compared with secretory phase endometrium, in proliferative endometrium we observed suggestive lower levels of *IGFBP1* and *IGF2* mRNA, and a higher frequency of positive glandular staining for the IR and pIGF1R/pIR proteins. In agreement with the current report, previous studies [37–39] observed higher expression of *IGF1* mRNA in premenopausal (irrespective of menstrual cycle phase) versus postmenopausal normal endometrium. On the other hand, *IGFBP1* and *IGFBP3* levels were higher in postmenopausal relative to premenopausal proliferative endometrium in the current study, which to our knowledge has not been previously published.

We observed a higher frequency of ER-positive staining in postmenopausal versus premenopausal proliferative endometrium and no difference in PR staining. The ER result contrasts with previous reports of similar [40–42] or lower frequencies of ER-positive staining [43] in postmenopausal as compared with premenopausal proliferative endometrium. The proportion of PR-positive glandular cell staining (~90 % positive) in postmenopausal women in the current study is in line with PR staining in a previous report [44]. We could not identify prior studies that had compared levels of the IR or IGF1R in postmenopausal versus premenopausal women. In general, different results across studies could be due to variability in IHC staining or scoring methods, or possible differences in the proportion of premenopausal endometrium sampled in early, mid, or late menstrual cycle phases as this information was unavailable in the current study. In particular, the latter issue may influence results pertaining to genes that are thought to play an important role in endometrial menstrual cycling (*IGF1*, *IGF2*, and *IGFBP1*); for example, *IGF1* mRNA is expressed preferentially in the mid-to-late proliferative and early secretory phases, while *IGF2* and *IGFBP1* are expressed in the mid-to-late secretory phase [45].

In contrast to earlier studies [42, 45–49], we did not observe differences in *IGF1* gene expression, or ER or PR IHC staining, by menstrual cycle phase. In the premenopausal endometrium, *IGF1* mRNA is thought to be estrogen-dependent, and increasing circulating estrogen levels during the proliferative phase may stimulate IGF-I production in the endometrial stromal cells that in turn induces the proliferation of endometrial glandular tissue [12, 45], reflected by the higher glandular pIGF1R/pIR protein levels in proliferative phase endometrium. Consistent with the current report, previous studies observed higher *IGF2* gene expression levels in secretory as compared with proliferative phase endometrium [45, 46, 50]. Furthermore, our observation of higher *IGFBP1* mRNA levels in the secretory versus proliferative phase endometrium supports the suggestion that higher circulating progesterone levels during the secretory phase may stimulate *IGFBP1* mRNA expression [45, 46]. We did not observe any variability in *IGFBP3* gene expression according to the menstrual cycle phase which contrasts with a previous study that reported higher *IGFBP3* mRNA levels in the secretory versus proliferative phase [45].

A potential limitation of our study was the small sample size. Due to the limited sample size, we were unable to adjust for age or other factors and therefore confounding may be an issue. The current study also did not assess how local tissue levels of the insulin/IGF and sex hormone components relate to circulating levels because the small sample size was a limiting factor in characterizing potentially complex relationships between the serum peptide levels and the tissue expression levels of mRNA and proteins. However, the novel data generated in the current study form a strong rationale and foundation for investigating the relationship of serum and tissue markers in future larger cohorts. Since a large number of statistical tests were carried out, it is possible that some of the results may be due to chance. Assay reproducibility was not assessed in our study; however, IHC staining and qPCR assays are routinely used and are considered reliable, and quality control measures were used for both methods (staining of positive and negative control tissues for IHC, RNA passed rigorous quality control assessment and assays were repeated in triplicate for qPCR). Women who have a hysterectomy may have different endometrial tissue or endometrial cancer risk factor distributions than the general population which may limit the generalizability of these findings. We did not validate our results in an independent premenopausal or postmenopausal population; therefore, the findings require confirmation in further studies. Nonetheless, our study had several important strengths including the systematic collection of fresh frozen and paraffin-embedded tissues from both premenopausal and postmenopausal women, and the linkage of epidemiologic and clinical information with tissue insulin/IGF and sex hormone expression levels which provides novel data on the impact of several established endometrial cancer risk factors on otherwise healthy endometrium.

In summary, we evaluated several major components of the insulin/IGF and sex hormone axes in normal endometrial tissues from women without cancer in order to describe their relationship with endometrial cancer risk factors to further knowledge on potential biological mechanisms. Among postmenopausal participants, we observed higher pIGF1R/pIR levels in endometrial tissues from diabetic versus non-diabetic women, which may reflect the impact of high circulating insulin levels on activation of these cancer-related receptors, and regular use versus no use of NSAIDs was associated with a higher expression of PR but not ER, which could be associated with a lower endometrial cancer risk. Thus, the current studies provide preliminary data pointing to mechanistic factors that may contribute to early events in the multistage process of endometrial carcinogenesis, with potential for early disease prevention or risk stratification.

## Electronic supplementary material

Below is the link to the electronic supplementary material.

Supplementary material 1 (DOCX 757 kb)

Supplementary material 2 (XLSX 45 kb)

Supplementary material 3 (XLSX 37 kb)

Supplementary material 4 (XLSX 9 kb)
