# Supplementary material for: Insulin/IGF and sex hormone axes in human endometrium and associations with endometrial cancer risk factors
Source: Cancer Causes Control. 2016 Apr 28;27:737–48. doi: 10.1007/s10552-016-0751-4 (PMC4870288; doi:10.1007/s10552-016-0751-4)
Supplement: Supplementary file 1 — Supplementary material 1 (DOCX 757 kb) [file 10552_2016_751_MOESM1_ESM.docx]

**Electronic Supplementary Material**

Article title. Insulin/IGF and Sex Hormone Axes in Human Endometrium and Associations with Endometrial Cancer Risk Factors

Journal name. Cancer Causes & Control

Author names. Merritt *et al.*

Affiliation and e-mail address of the corresponding author. Melissa A. Merritt, Department of Epidemiology and Biostatistics, School of Public Health, Imperial College London, St Mary’s Campus, Norfolk Place, London, W2 1PG, UK.

E-mail: m.merritt@imperial.ac.uk

**Supplementary Tables (1-2) and Figures (1-2)**

**Supplementary Table 1** Antibodies and experimental conditions used in immunohistochemistry

| **Antigen** | **Manufacturer** | **Catalog #** | **Host** | **Antigen retrieval** | **Dilution** | **Incubation time** |
| --- | --- | --- | --- | --- | --- | --- |
| ERα | DAKO | M7047 | Monoclonal Mouse | Citrate pH 6 | 1/35 | 30min at RT |
| PR | DAKO | M3569 | Monoclonal Mouse | Citrate pH 6 | 1/50 | 30min at RT |
| IGF1R | Cell Signaling | #3027 | Polyclonal Rabbit | Citrate pH 6 | 1/150 | Overnight at 4°C |
| pIGF1R/pIR | Cell Signaling | #3021 | Polyclonal Rabbit | Citrate pH 6 | 1/50 | Overnight at 4°C |
| IR | Santa Cruz | Sc-573442 | Monoclonal Mouse | Citrate pH 6 | 1/50 | Overnight at 4°C |
| PTEN | Cell Signaling | #9188 | Monoclonal Rabbit | EDTA pH 8.0 | 1/100 | Overnight at 4°C |

**Abbreviations:** room temperature (RT)

**Supplementary Table 2** Real-time PCR primers

| Analyte | Genbank Accession | Forward Primer  (5’- ) | Reverse Primer  (5’ - ) |
| --- | --- | --- | --- |
| *IGF-I* | NM_001111284.1 | GCTCTTCAGTTCGTGTGTGGA | CGACTGCTGGAGCCATACC |
| *IGF-II* | NM_000612.4 | ACCGTGCTTCCGGACAACT | TGGACTGCTTCCAGGTGTCA |
| *IGFBP1* | NM_000596.2 | AATGGAAGGAGCCCTGCCGAA | CTGATGTCTCCTGTGCCTTGGCT |
| *IGFBP3* | NM_001013398.1 | CACTGAATCACCTGAAGTTCCTC | AGGGCGACACTGCTTTTTCTT |
| *PPIB* | NM_00942.4 | AAGTCACCGTCAAGGTGTATTTT | GATCACCCGGCCTACATCTTC |

**Supplementary Figure 1.** **Insulin/IGF axis protein expression in endometrium in relation to menstrual cycle phase**.

| **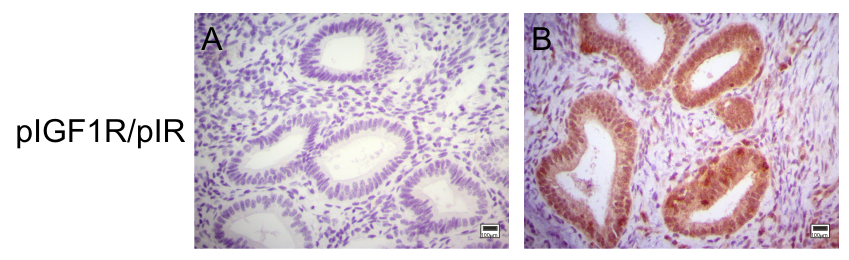** |
| --- |

**A**, pIGF1R/pIR glandular cytoplasmic staining is negative in this representative tissue sample of secretory phase endometrium from a premenopausal patient. **B**, pIGF1R/pIR glandular and nuclear cytoplasmic staining is positive in this representative tissue sample of proliferative phase endometrium from a premenopausal patient. Pictured images are of equal magnification (400x) and scale (the 100 µm scale bar is shown).

**Supplementary Figure 2.** **Gene expression of IGF axis genes in premenopausal endometrium in relation to menopausal cycle phase**. Gene expression values (normalized to *PPIB*) as detected by qPCR are pictured. Box and whisker plots depict the median (line), interquartile range (box) and error bars demonstrate the full range of the data.

| **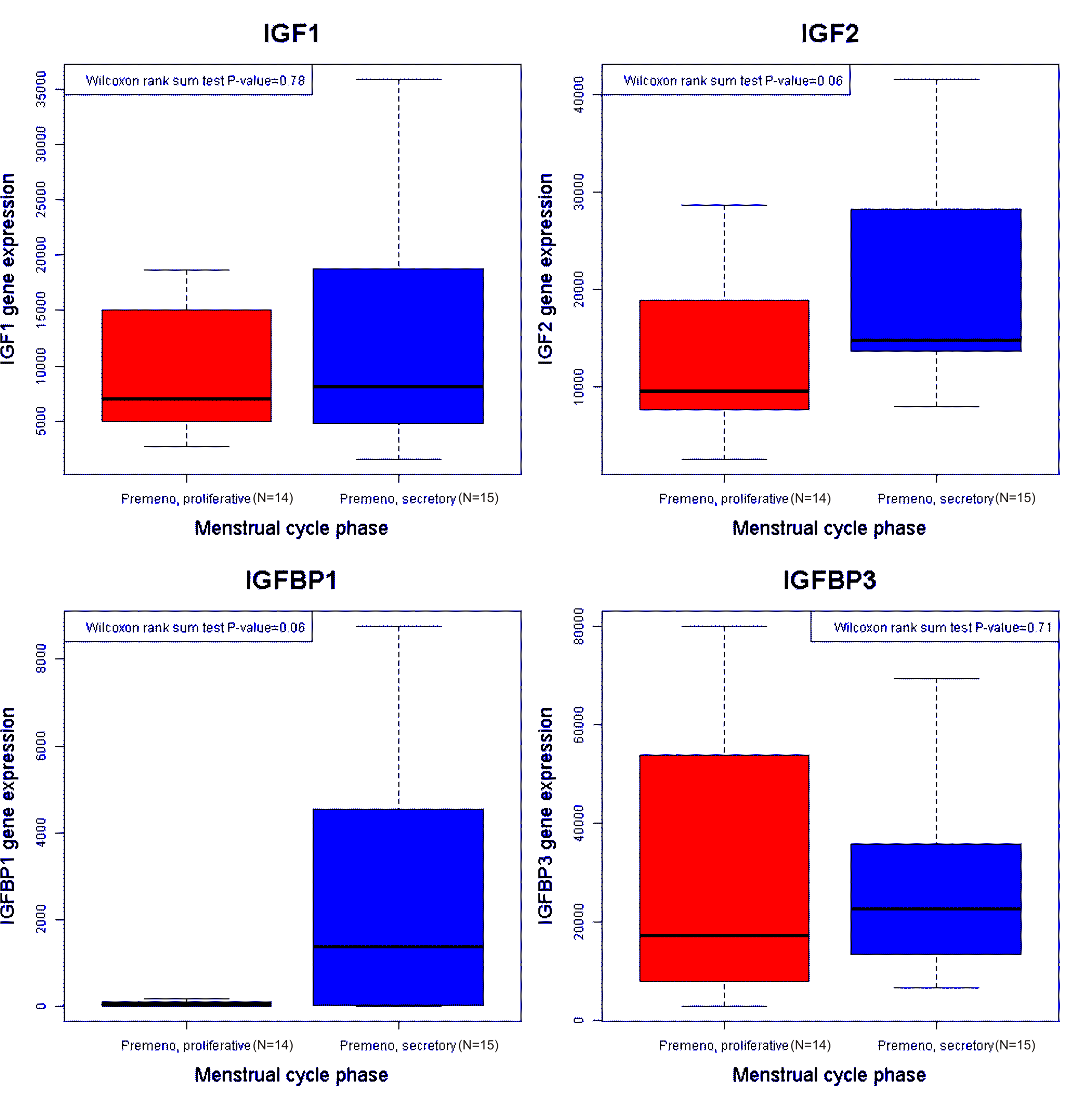** |
| --- |
